# Inverse association of plasma IL-13 and inflammatory chemokines with lung function impairment in stable COPD: a cross-sectional cohort study

**DOI:** 10.1186/1465-9921-8-64

**Published:** 2007-09-14

**Authors:** Janet S Lee, Matthew R Rosengart, Venkateswarlu Kondragunta, Yingze Zhang, Jessica McMurray, Robert A Branch, Augustine MK Choi, Frank C Sciurba

**Affiliations:** 1 Division of Pulmonary, Allergy, and Critical Care Medicine, Department of Medicine, University of Pittsburgh, Pittsburgh, PA, 15213, USA; 2 Division of Trauma/General Surgery, Department of Surgery, University of Pittsburgh, Pittsburgh, PA, 15213, USA; 3 Division of Clinical Pharmacology, Department of Medicine, University of Pittsburgh, Pittsburgh, PA, 15213, USA

## Abstract

**Background:**

Chronic obstructive pulmonary disease (COPD) is a heterogeneous syndrome characterized by varying degrees of airflow limitation and diffusion impairment. There is increasing evidence to suggest that COPD is also characterized by systemic inflammation. The primary goal of this study was to identify soluble proteins in plasma that associate with the severity of airflow limitation in a COPD cohort with stable disease. A secondary goal was to assess whether unique markers associate with diffusion impairment, based on diffusion capacity of carbon monoxide (DLCO), independent of the forced expiratory volume in 1 second (FEV_1_).

**Methods:**

A cross sectional study of 73 COPD subjects was performed in order to examine the association of 25 different plasma proteins with the severity of lung function impairment, as defined by the baseline measurements of the % predicted FEV_1 _and the % predicted DLCO. Plasma protein concentrations were assayed using multiplexed immunobead-based cytokine profiling. Associations between lung function and protein concentrations were adjusted for age, gender, pack years smoking history, current smoking, inhaled corticosteroid use, systemic corticosteroid use and statin use.

**Results:**

Plasma concentrations of CCL2/monocyte chemoattractant protein-1 (CCL2/MCP-1), CCL4/macrophage inflammatory protein-1β (CCL4/MIP -1β), CCL11/eotaxin, and interleukin-13 (IL-13) were inversely associated with the % FEV_1_. Plasma concentrations of soluble Fas were associated with the % DLCO, whereas CXCL9/monokine induced by interferon-γ (CXCL9/Mig), granulocyte- colony stimulating factor (G-CSF) and IL-13 showed inverse relationships with the % DLCO.

**Conclusion:**

Systemic inflammation in a COPD cohort is characterized by cytokines implicated in inflammatory cell recruitment and airway remodeling. Plasma concentrations of IL-13 and chemoattractants for monocytes, T lymphocytes, and eosinophils show associations with increasing severity of disease. Soluble Fas, G-CSF and CXCL9/Mig may be unique markers that associate with disease characterized by disproportionate abnormalities in DLCO independent of the FEV_1_.

## Background

Chronic obstructive pulmonary disease (COPD), while defined by the presence of incompletely reversible airflow obstruction, represents a syndrome of various physiologic impairments [1,2]. COPD is also defined by "an abnormal inflammatory response to noxious stimuli" [1,2], and increasing evidence suggests that COPD is a disease characterized by both local and systemic inflammation [3].

The best characterized systemic marker is C-reactive protein (CRP) [3,4], but its lack of specificity provides little insight into potential mechanisms underlying the systemic inflammation characterizing COPD. We hypothesize that this systemic inflammation may be further characterized by examining associations between physiologic indices of lung function impairment and members of various classes of soluble proteins. To date, studies examining the association between a wide range of soluble proteins in plasma and severity of lung function impairment during stable COPD are lacking. This is due, in part, to the limited amount of sample that can be obtained from subjects at any given time.

We conducted an exploratory analysis to determine the associations between increasing physiologic severity of COPD, as defined by the % predicted FEV_1 _or % DLCO, during stable disease and plasma concentrations of 25 different cytokines and growth factors. We adjusted for current cigarette smoking and corticosteroid use because others have shown that these factors may be potential modifiers of systemic inflammation in this cohort [5-7]. We also adjusted for variables such as gender, age, statin use, and pack years smoking that may influence cytokine levels. This analysis represents an important, initial stage in identifying candidate plasma proteins for future prospective, longitudinal studies and one that utilizes a new technique to assay for multiple cytokines at a given time.

## Methods

### Patient selection

Seventy-three individuals enrolled in the Emphysema/COPD Research Center (ECRC) of the University of Pittsburgh gave informed consent for the study. Inclusion criteria included clinically stable COPD at the time of the examination, tobacco exposure of at least 10 pack years, and no clinical diagnosis of rheumatologic, infectious or other systemic inflammatory disease. Exclusion criteria included dominant restrictive spirometric impairment, a significant allergic history, completely reversible airflow obstruction or a history of clinical asthma. The study was approved by the University of Pittsburgh Institutional Review Board.

### Pulmonary function measurements

Spirometry was performed on 73 subjects using standard methodology at the time of entry into the study [8-10]. Fifty-three subjects also had single breath carbon monoxide diffusing capacity using standard methodology [11]. Standard reference equations for % FEV_1 _and % DLCO were used [12,13].

### Plasma marker measurements

Plasma samples were obtained from subjects upon enrollment into the ECRC registry. Blood was collected into acid citrate dextrose (ACD) cell preparation tubes (CPT tubes). Samples were processed immediately, and plasma was isolated and stored immediately at -80°C until analyzed. A detailed methods of the multiplex assay performed at the University of Pittsburgh Cancer Institute Luminex Core Facility has been previously described [14]. We have previously used a multiplex immuno-bead assay system (Luminex, Austin, TX, USA) to assay multiple systemic cytokine concentrations using both mouse and human plasma samples [15]. Reproducibility of cytokine signals for inter-individual comparisons using stimulated plasma samples has been previously demonstrated using the multiplex format [16]. Four sets of plates were used to assay a total of 28 cytokines and inflammatory markers: Set 1) Twenty-three cytokines in multiplex format (Biosource Invitrogen, Camarillo, CA); Set 2) EGFR, Fas, and FasL analytes in multiplex format (University of Pittsburgh Luminex Core Facility, Pittsburgh, PA); Set 3) CRP concentrations (LINCO Research, St. Charles, Missouri); Set 4) MPO concentrations (LINCO Research, St. Charles, Missouri). All samples were assayed simultaneously to minimize day-to-day variability (Table 1).

**Table 1:** Detectability of plasma marker concentrations

Classification	Plasma marker	Mean (pg/mL) + SE	LLD* (pg/mL)	below LLD (%)
**Apoptosis**	Fas	74 ± 3	1.3	0
	FasL	68 ± 4	6.5	0
**Acute phase**	CRP	6268332 ± 1124813	78	0
	MPO	4381 ± 589	13	3
**Chemokines**	CCL2/MCP -1	202 ± 6	10	0
	CCL3/MIP-1α	161 ± 33	6.8	5
	CCL4/MIP-1β	115 ± 20	1.3	1
	CCL5/RANTES	5249 ± 398	8.2	0
	CCL11/eotaxin	76 ± 3	2.3	0
	CXCL8/IL-8	11 ± 0.4	6.1	0
	CXCL9/Mig	1163 ± 87	35	0
**T_H _Related Cytokines**	IFN-γ	55 ± 7	2.1	8
	IL-2	103 ± 23	2.6	23
	IL-2R^†^	344 ± 28	39	0
	IL-4	15 ± 3	0.6	25
	IL-13	98 ± 7	8.2	11
**Inflammation**	TNF-α	55 ± 7	5.3	0
	TNFRI^†^	1352 ± 95	36	0
	TNFRII^†^	3231 ± 138	39	0
	IL-1β	72 ± 17	8.5	45
	IL-6	19 ± 4	0.4	1
	IL-10	0.3 ± 0.08	0.2	96
**Growth Factors**	EGF	19 ± 1.5	2.5	0
	EGFR^†^	19769 ± 434	13.5	0
	FGFβ	NE^‡^	NE^‡^	NE^‡^
	G-CSF	2496 ± 180	379	0
	HGF	196 ± 9	2.8	0
	VEGF	NE^‡^	NE^‡^	NE^‡^

*LLD, Lower Limit of Detection

^†^For clarity, the soluble receptors are grouped with their respective ligand

^‡^NE, Not Evaluable

Selection of specific cytokines in the study was based upon two main criteria: (1) availability of reagent using the Luminex platform, and (2) prior published data to suggest biological plausibility of a cytokine or soluble protein in either systemic or local inflammation observed in COPD. We chose six broad classes of soluble proteins and measured representative markers (Table 1). Apoptosis-related proteins included soluble Fas, FasL, soluble TNFRI and TNFRII [17-19]. Acute phase reactants included C-reactive protein (CRP) [4] and Myeloperoxidase (MPO) [20]. Representative chemokines included CCL2/MCP-1, CCL3/MIP-1α and CCL4/MIP-1β [21], CCL5/RANTES [22], CCL11/eotaxin [23], CXCL8/IL-8 [24,25], and CXCL9/Mig [26]. T_H _related cytokines were also of considerable interest, given recent findings regarding the role of the T_H _phenotype in COPD [27-29]. Representative T_H1 _and T_H2 _cytokines interferon-gamma (IFN-γ), interleukin-2 (IL-2) and its soluble receptor IL-2R, interleukin-4 (IL-4), and IL-13 were chosen on this basis. Inflammation related proteins included TNF-α [30,31], soluble TNFR1 and TNFRII [30,31], IL-1β [32], IL-6 [32], and IL-10 [28]. Growth factors included epidermal growth factor (EGF) and its soluble receptor epidermal growth factor receptor (EGFR) [33-35], fibroblast growth factor beta (FGFβ) [36,37], granulocyte-colony stimulating factor (G-CSF)[38], hepatocyte growth factor (HGF) [39], and vascular endothelial growth factor (VEGF) [40].

Standard curves were generated according to the manufacturer's instructions. Goodness of fit for standard curves was determined by the standards recovery method and performed by calculating the following equation for the concentration of each standard: (observed concentration/expected concentration) × 100. Concentrations for the unknown samples were calculated based upon a 5 parametric curve fitting program (Bio-Rad Laboratories, Hercules, CA). The 5 parametric curve fitting program yields extrapolated values beyond the concentrations for a given standard curve as determined by conventional linear regression, and is the preferred mathematical modeling for multiplex immunoassays [41,42]. This provided a greater detectable range of observed concentrations, and was particularly useful for analytes where plasma concentrations of samples were uniformly low.

We defined the lower limit of detection (LLD) for each analyte as the lowest observed concentration in pg/mL. This was, in some instances, an extrapolated value that was lower than the lowest standard curve concentration. Unknown sample concentrations, below the LLD for a given analyte, were assigned a value set just below the LLD using the following equation: undetectable value = LLD of analyte/squared root 2. This method of assigning a value for unknown sample concentrations with undetectable levels has been previously used to examine the relationship of impaired lung function to circulating levels of C-reactive protein and fibrinogen [4]. This allowed for the inclusion of all samples in our analysis, with data shown in Table 1.

### Statistical analysis

We performed univariate and multivariate linear regression analysis to test the association between the concentration of each plasma cytokine and the physiologic indices of interest: percent (%) predicted FEV_1 _and the % predicted DLCO. The dependent variable of interest, plasma cytokine concentration, was not normally distributed; thus, values were log transformed to meet the assumption of normality for linear regression. Standard regression diagnostics were performed to ensure the assumptions for linear regression were met. Covariates previously published as associated with the outcomes of interest (e.g. current smoking and corticosteroid use) were identified *a priori *and also included [5-7]. We also included variables presumed to alter cytokine values: age, gender, statin use, and pack year smoking history. Statistical significance was determined at a p-value < 0.05. We did not attempt to adjust for multiple comparisons as our emphasis, being exploratory, was to minimize a Type I error and any adjustment could potentially miss real differences within the scope of this modest sample size [43]. SAS 8.2 (SAS Institute Inc., Cary, NC) and STATA 9.0 (Stata Corporation, College Station, Texas) softwares were used for analysis.

## Results

### Subject demographics

Seventy-three individuals were recruited for analysis. Table 2 shows the subject demographics for each Global initiative for Chronic Obstructive Lung Disease (GOLD) classification. The prevalence of cigarette smoking decreased and the use of inhaled or systemic corticosteroids increased with more severe airflow obstruction.

**Table 2:** Demographics, comparison of subjects by GOLD classification

	GOLD 0	GOLD 1	GOLD 2	GOLD 3	GOLD 4	Total
Sample size	5	8	21	20	19	73
Age, years*	61 (2)	59 (2)	64 (2)	67 (2)	60 (2)	63 (1)
Sex, M/F	3/2	5/3	12/9	13/7	9/10	42/31
Pack years*	37 (4)	57 (8)	60 (7)	53 (4)	47 (4)	53 (3)
Current smokers (%)	2 (40)	4 (50)	7 (33)	5 (25)	1 (5)	19 (26)
ICS use (%)	0 (0)	1 (13)	7 (33)	12 (60)	16 (84)	36 (49)
SCS use (%)	0 (0)	0 (0)	0 (0)	2 (10)	3 (16)	5 (7)
% FEV_1 _*	87 (4)	91 (3)	66 (2)	39 (1)	21 (1)	51 (3)
FEV_1_/FVC*	77(2)	63 (2)	55 (2)	37 (2)	28 (1)	45 (2)
% DLCO* ^†^	68 (4)	58 (7)	62 (4)	37 (2)	25 (1)	46 (3)

ICS, inhaled corticosteroids; SCS, systemic corticosteroids

*Data are presented as mean (SEM)

^†^DLCO % predicted measurements not available for 1 subject in GOLD 0, 2 subjects in GOLD 1, 6 subjects in GOLD 2, 6 subjects in GOLD 3, 5 subjects in GOLD 4.

Fifty-three of the 73 individuals from the cohort received DLCO measurements (Table 3). We addressed the potential for selection bias by comparing the patient characteristics of those with and without DLCO measurements. There was no significant difference between those with and those without DLCO measurements for any of the patient characteristics.

**Table 3:** Demographics, comparison of subjects with and without % DLCO measurements

	Subjects with DLCO	Subjects without DLCO	p-value
Sample size	53	20	-
Age, years*	64 (1)	61 (2)	0.16
Sex, M/F	32/21	10/10	0.43
Pack years*	54 (3)	49 (5)	0.49
Current smokers (%)	15 (28)	4 (20)	0.48
ICS use (%)	24 (45)	12 (60)	0.27
SCS use (%)	3 (6)	2 (10)	0.52
% FEV_1 _*	51 (4)	51 (6)	0.97
FEV_1_/FVC*	45 (2)	46 (4)	0.89

*Data are presented as mean (SEM).

### Detectability of plasma protein concentrations

Twenty-eight markers from 6 classes of soluble proteins were originally measured. The mean plasma concentrations in pg/mL are depicted in Table 1. Sixteen of 28 proteins showed detectable concentrations for all samples (Table 1). Ten of 28 proteins were below the detectable range for some samples (MPO, CCL3/MIP-1α, CCL4/MIP-1β, IFN-γ, IL-2, IL-4, IL-13, IL-1β, IL-6, IL-10). None of the samples were above the detectable range for any of the proteins measured. IL-10 concentrations were undetectable in virtually all patients (70/73, 96%), and standard curves generated for FGFβ and VEGF were consistently poor. Thus, IL-10, FGFβ, and VEGF were excluded from further analysis, and a total of 25 cytokines were assessed for an association with severity of lung function impairment.

### Association between systemic cytokines and FEV1

In univariate analyses, increasing concentrations of T helper (T_H_) related cytokines interferon-γ (IFN-γ), interleukin-2 (IL-2), interleukin-4 (IL-4) and IL-13 were associated with increasing severity of airflow obstruction, as characterized by decreasing % predicted FEV_1 _(Table 4). Increasing concentration of the monocyte and T lymphocyte chemokine CCL4/MIP-1β was also associated with increasing severity of airflow obstruction (Table 4).

**Table 4:** Association between plasma marker concentrations and % FEV1, unadjusted

Classification	Plasma marker	β^†^	95% CI	p^‡^
**Apoptosis**	Fas	0.003	-0.001, 0.01	0.12
	FasL	0.001	-0.004, 0.01	0.67
**Acute phase**	CRP	-0.01	-0.02, 0.003	0.19
	MPO	0.0004	-0.01, 0.01	0.93
**Chemokines**	CCL2/MCP -1	-0.002	-0.004, 0.001	0.14
	CCL3/MIP-1α	-0.004	-0.02, 0.01	0.53
	CCL4/MIP-1β	-0.01	-0.02, -0.003	< 0.01
	CCL5/RANTES	-0.005	-0.01, 0.002	0.18
	CCL11/eotaxin	-0.003	-0.006, 0.0004	0.09
	CXCL8/IL-8	-0.002	-0.01, 0.001	0.18
	CXCL9/Mig	-0.01	-0.01, 0.001	0.09
**T**_H_**Related Cytokines**	IFN-γ	-0.01	-0.03, -0.001	0.04
	IL-2	-0.02	-0.04, -0.002	0.03
	IL-2R^§^	-0.005	-0.01, 0.0003	0.06
	IL-4	-0.02	-0.03, -0.003	0.02
	IL-13	-0.01	-0.02, -0.0004	0.04
**Inflammation**	TNF-α	-0.01	-0.02, 0.002	0.11
	TNFRI^§^	-0.001	-0.01, 0.004	0.72
	TNFRII^§^	-0.001	-0.01, 0.004	0.83
	IL-1β	-0.01	-0.02, 0.01	0.47
	IL-6	-0.003	-0.01, 0.01	0.61
	IL-10	-0.001	-0.01, 0.004	NE*
**Growth Factors**	EGF	-0.01	-0.01, 0.001	0.09
	EGFR^§^	0.001	-0.001, 0.003	0.25
	FGFβ	NE*	NE*	NE*
	G-CSF	-0.003	-0.01, 0.002	0.28
	HGF	-0.002	-0.01, 0.001	0.20
	VEGF	NE*	NE*	NE*

^†^β = regression co-efficient

^‡^p = p-value

^§^For clarity, the soluble receptors are grouped with their respective ligand

*NE, Not Evaluable

We did not observe significant associations between plasma CRP concentrations and the % predicted FEV_1_. We explored the effect of inhaled corticosteroids on the relationship between CRP and the % FEV_1_because of previous findings that inhaled corticosteroids can suppress systemic CRP levels [6]. In contrast to other cytokines examined, we noted interaction between corticosteroids with CRP concentrations (p = 0.05). An overall association was not observed between increasing plasma CRP with increasing severity of airflow limitation because the magnitude of the difference in CRP concentration across % FEV_1 _was diminished in those with corticosteroid use as compared to those without (data not shown).

### Multivariate model of the association between systemic cytokines and FEV_1_

After adjusting for age, gender, pack years smoking history, current smoking, inhaled corticosteroid use, systemic corticosteroid use and statin use, three of the seven chemokines examined were significantly associated with % FEV_1 _(Table 5). Increasing concentrations of chemokines CCL4/MIP-1β, CCL2/MCP-1, and CCL11/eotaxin were associated with increasing severity of airflow obstruction. Of the 4 T_H _related cytokines that showed associations with % FEV1 in univariate analysis (Table 4), only IL-13 remained significant (Table 5). Thus, CCL4/MIP-1β and IL-13 showed inverse associations with % FEV1 both by univariate and multivariate analysis.

**Table 5:** Association between plasma markers and % FEV_1_, adjusted*

Analyte	β^†^	95% CI	p^‡^
CCL2/MCP -1	-0.003	-0.005, -0.001	0.02
CCL4/MIP-1β	-0.01	-0.02, -0.001	0.04
CCL11/eotaxin	-0.005	-0.01, -0.002	0.004
CXCL9/Mig	-0.01	-0.02, 0.0003	0.06
EGF	-0.005	-0.01, 0.004	0.24
IFN-γ	-0.01	-0.03, 0.002	0.08
IL-2	-0.02	-0.03, 0.004	0.12
IL-2R	-0.005	-0.01, 0.002	0.15
IL-4	-0.02	-0.03, 0.001	0.07
IL-13	-0.01	-0.02, -0.001	0.04

*Adjusted for current smoking, pack years, ICS use, SCS use, statin use, gender and age.

^†^β = regression co-efficient

^‡^p = p-value

### Association between systemic cytokines and DLCO

We examined the association between systemic cytokines and the % predicted DLCO (Table 6). Increasing concentrations of chemokines CCL4/MIP -1β, CC chemokine ligand 5/Regulated on Activation Normal T cell Expressed and Secreted (CCL5/RANTES), CXC chemokine ligand 8/interleukin 8 (CXCL8/IL-8), and CXCL9/Mig were associated with increasing severity of diffusion impairment, as characterized by decreasing % predicted DLCO. Similar to FEV_1_, T_H _related cytokines IFN-γ, IL-2, IL-4 and IL-13 showed inverse associations with the % predicted DLCO. We also observed that increasing concentrations of TNF-α, epidermal growth factor (EGF) and G-CSF associated with increasing severity of diffusion impairment. This is in contrast to soluble Fas where lower concentrations were associated with increasing severity of diffusion impairment. Systemic markers such as CRP, IL-6 and MPO did not show significant associations with the % predicted DLCO.

**Table 6:** Association between plasma marker concentrations and % DLCO, unadjusted

Classification	Plasma marker	β^†^	95% CI	p^‡^
**Apoptosis**	Fas	0.01	0.001, 0.01	0.01
	FasL	-0.0004	-0.007, 0.006	0.89
**Acute phase**	CRP	-0.01	-0.02, 0.01	0.33
	MPO	-0.005	-0.01, 0.004	0.30
**Chemokines**	CCL2/MCP -1	-0.003	-0.01, 0.0004	0.09
	CCL3/MIP-1α	0.001	-0.02, 0.02	0.92
	CCL4/MIP-1β	-0.02	-0.03, -0.001	0.04
	CCL5/RANTES	-0.01	-0.02, -0.002	0.02
	CCL11/eotaxin	-0.002	-0.01, 0.002	0.31
	CXCL8/IL-8	-0.005	-0.01, -0.002	< 0.01
	CXCL9/Mig	-0.01	-0.02, -0.004	< 0.01
**T**_H_**Related Cytokines**	IFN-γ	-0.03	-0.04, -0.01	< 0.001
	IL-2	-0.04	-0.06, -0.02	< 0.01
	IL-2R^§^	-0.005	-0.01, 0.002	0.18
	IL-4	-0.03	-0.06, -0.01	< 0.01
	IL-13	-0.02	-0.03, -0.01	< 0.001
**Inflammation**	TNF-α	-0.02	-0.03, -0.005	< 0.01
	TNFRI^§^	0.001	-0.01, 0.01	0.79
	TNFRII^§^	0.001	-0.004, 0.005	0.84
	IL-1β	-0.01	-0.03, 0.004	0.13
	IL-6	-0.01	-0.02, 0.004	0.17
	IL-10	-0.01	-0.01, 0.003	NE*
**Growth Factors**	EGF	-0.01	-0.02, 0.0001	0.05
	EGFR^§^	0.002	-0.001, 0.005	0.12
	FGFβ	NE*	NE*	NE*
	G-CSF	-0.01	-0.02, -0.002	0.02
	HGF	-0.005	-0.01, 0.001	0.08
	VEGF	NE*	NE*	NE*

^†^β = regression co-efficient

^‡^p = p-value

^§^For clarity, the soluble receptors are grouped with their respective ligand

*NE, Not Evaluable

### Multivariate model of the association between systemic cytokines and DLCO

We further examined the relationship between plasma concentrations of inflammatory markers and the % predicted DLCO, adjusting for the % FEV_1_, age, gender, pack years smoking history, current smoking, inhaled corticosteroid use, systemic corticosteroid use and statin use (Table 7). The inverse associations between % DLCO and CXCL9/Mig, G-CSF, and IL-13 remained significant. The association between soluble Fas and % DLCO also remained significant.

**Table 7:** Association between plasma markers and % DLCO, adjusted*

Analyte	β^†^	95% CI	p^‡^
CCL2/MCP-1	-0.001	-0.01, 0.003	0.58
CCL4/MIP-1β	0.004	-0.02, 0.03	0.76
CCL5/RANTES	-0.01	-0.03, 0.003	0.11
CCL11/eotaxin	-0.005	-0.01, 0.002	0.15
CXCL8/IL-8	-0.004	-0.01, 0.002	0.18
CXCL9/Mig	-0.02	-0.03, -0.002	0.02
EGF	-0.01	-0.03, 0.01	0.29
Fas	0.01	0.003, 0.02	0.01
G-CSF	-0.01	-0.02, -0.0001	0.05
HGF	-0.0001	-0.01, 0.01	0.99
IFN-γ	-0.02	-0.05, 0.01	0.11
IL-2	-0.03	-0.06, 0.01	0.11
IL-2R	0.01	-0.01, 0.02	0.46
IL-4	-0.02	-0.05, 0.02	0.30
IL-13	-0.02	-0.03, -0.002	0.03
TNF-α	-0.01	-0.03, 0.003	0.11

*Adjusted for % FEV1, current smoking, pack years, ICS use, SCS use, statin use, gender and age.

^†^β = regression co-efficient

^‡^p = p-value

### IL-13 and Bronchodilator Reversiblity

**Figure 1:**
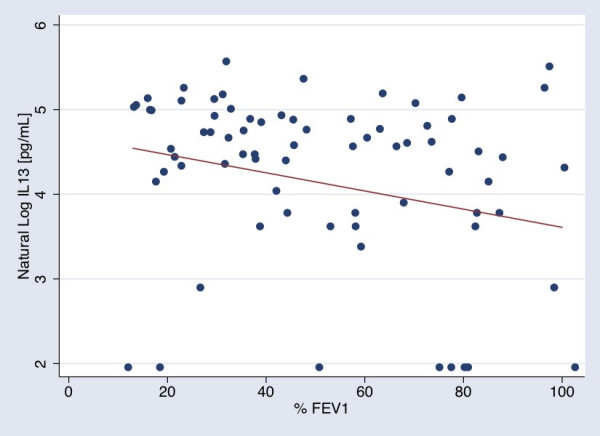
The relationship between natural log (LN) IL-13 concentrations in pg/mL and % predicted FEV1. The line was calculated using conditional standardization of the regression results for a patient with mean and modal values for the covariates in the model. The standardized line thus represents the relationship between IL-13 and FEV_1 _for a man, age 63, who does not currently smoke, with mean pack year smoking history of 52.5 years, who is not on statins or systemic steroids, but is on inhaled steroids (β = -0.01).

**Figure 2:**
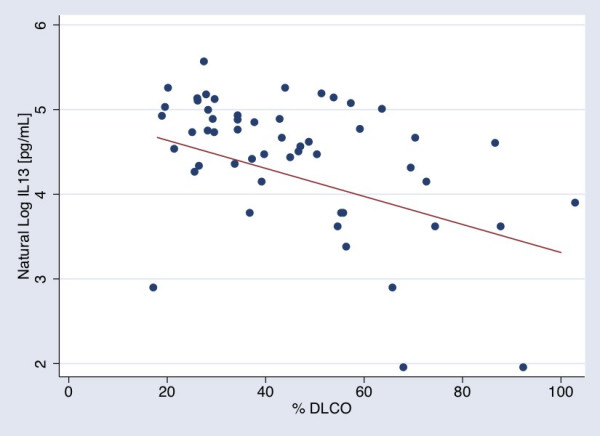
The relationship between natural log (LN) IL-13 concentrations in pg/mL and % predicted DLCO. The line was calculated using conditional standardization of the regression results for a patient with mean and modal values for the covariates in the model. The standardized line thus represents the relationship between IL-13 and DLCO for a man, age 63, with a FEV_1 _of 51 % predicted, who does not currently smoke, with mean pack year smoking history of 52.5 years, who is not on statins or systemic steroids, but is on inhaled steroids (β = -0.02).

Of the 25 cytokines examined, increasing plasma concentrations of IL-13 showed inverse relationships with both % FEV_1 _and % DLCO (Figures 1 &2). We tested the possibility that a subset of the population with bronchodilator reversibility may account for the inverse association between IL-13 and % FEV_1_. Of those subjects with available information, 12 out of the 73 subjects in the cohort met ATS/ERS task force definition for bronchodilator response [44]. Excluding these 12 individuals did not alter the association between IL-13 and % FEV_1 _(β = -0.01, p = 0.01). An additional 15 out of the 73 subjects did not have bronchodilator reversibility testing at the time of study entry, although 10 of these subjects had emphysema by CT scan and/or abnormally low % predicted DLCO. Further excluding these 15 individuals with unknown bronchodilator response from the cohort, the point estimates for the association between IL-13 and % FEV_1 _in the remaining 46 subjects was essentially unchanged but did not reach significance due to greater variation (β = -0.01, p = 0.06).

## Discussion

We examined the association between 25 different plasma markers of inflammation and two physiologic parameters of COPD in a well-defined clinical population. The main observation was that increasing severity of airflow limitation, as defined by the % FEV_1_, was associated with increasing systemic concentrations of IL-13, and the inflammatory chemokines CCL2/MCP-1, CCL4/MIP-1β, and CCL11/eotaxin after adjusting for age, gender, pack years smoking history, current smoking, inhaled corticosteroid use, systemic corticosteroid use and statin use. Furthermore, increasing severity of diffusion impairment, as defined by the % DLCO, was associated with increasing IL-13, CXCL9/Mig, and G-CSF concentrations and decreasing soluble Fas concentrations.

In both univariate and multivariate analysis, increasing plasma concentration of the T helper 2 (T_H2_) type cytokine IL-13 was associated with increasing severity of airflow obstruction, suggesting that IL-13 may be an important mediator in human COPD. The association between increasing IL-13 concentrations and increasing severity of airflow obstruction could not be accounted for by a subset of the cohort with bronchodilator reversibility. This finding suggests that the association is unlikely due to misclassification of asthmatic patients in our COPD cohort.

IL-13 is implicated in airway mucin production and airway inflammation [45,46]. IL-13 has been previously shown to induce mucous metaplasia and chemokine expression in animal models of allergic airway inflammation and emphysema [47,48]. Others have recently shown that both CD4^+ ^and CD8^+ ^T cells in the bronchoalveolar lavage fluid of COPD patients expressed significantly higher percentages of IL-13 than smokers with normal lung function and never smokers [28]. Similar to our findings, these authors showed a negative correlation between intracellular IL-13 and % FEV_1_.

Three of seven chemokines tested were associated with increasing severity of airflow obstruction: CCL2/MCP-1, CCL4/MIP-1β, and CCL11/Eotaxin. In addition, CXCL9/Mig was associated with increasing severity of diffusion impairment. These chemokines recruit primarily monocytes, T lymphocytes, and eosinophils, inviting the possibility that soluble proteins that promote inflammatory cell recruitment contribute to the low-grade systemic inflammation observed in COPD. CCL2/MCP-1 recruits monocytes and T lymphocytes expressing the receptor CCR2 [49], and increased concentrations of this chemokine have been reported in induced sputum, BAL and lung tissue of COPD individuals [38,50]. CCL4/MIP-1β can recruit CCR5 expressing monocytes and T lymphocytes [49]. Our data corroborates findings showing a negative correlation between CCL4/MIP-1β concentrations in the BAL from patients with chronic bronchitis and the % FEV_1 _[21]. CCL11/Eotaxin is involved in eosinophil recruitment [51], and CCL11/eotaxin concentrations are increased in the sputum of patients with exacerbations of chronic bronchitis [23]. However, some COPD patients with stable disease also show airway eosinophilic inflammation [52].

A secondary goal of this study was to explore whether systemic cytokines are associated with severity of diffusion impairment, the physiologic parameter that corresponds best to the loss of alveolar-capillary bed surface area in emphysema. In the smaller cohort that received DLCO measurements, it is interesting that CXCL9/Mig concentration was inversely associated with % DLCO. CXCL9/Mig recruits CXCR3 expressing T lymphocytes [49]. Saetta and colleagues have previously shown increased numbers of CXCR3 expressing T lymphocytes in peripheral airways of COPD patients [53]. Upon stimulation with CXCL9/Mig, CD14^+ ^CXCR3^+ ^macrophages of human emphysematous lungs can increase metalloproteinase production in vitro [26]. Thus, recent findings suggest a potential link between this chemokine and the pro-elastolytic environment of emphysema.

Increasing concentrations of plasma G-CSF are also associated with increasing severity of diffusion impairment. G-CSF is involved in neutrophil mobilization and survival [54], however its role in COPD is not yet known. There are increased numbers of granulocytes in the sputum and BAL [38] in addition to small airways [55] of COPD patients, leading others to speculate that granulocyte survival in the lungs may be enhanced in COPD by mediators such as G-CSF [38].

Another molecule identified is soluble Fas. Decreasing concentrations of soluble Fas are associated with increasing severity of diffusion impairment. Soluble Fas, a result of alternative mRNA splicing, inhibits apoptosis by competitively binding FasL and preventing its interaction with the membrane bound Fas receptor [56,57]. The relationship between systemic levels of soluble Fas and COPD is unclear, as other smaller studies have shown variable findings of either elevation or no difference compared with controls [17-19]. Our results suggests that a systemic imbalance of the anti-apoptotic factor soluble Fas occurs in the setting of a pro-apoptotic environment of the lungs in COPD.

The limitations of this present study include the size of the cohort and its cross-sectional nature. The modest size, particularly the number of subjects with milder lung function impairment (GOLD 0–1 stages), may limit the ability to detect significant associations between systemic markers and lung function impairment. Furthermore, we included age, gender, pack years smoking history, current smoking, inhaled corticosteroid use, systemic corticosteroid use and statin use in the multivariate model. It is uncertain whether adjustment for these covariates is appropriate. Thus, we present both univariate and multivariate analysis. We also recognize that the observed associations between plasma concentrations of a protein and lung function severity do not necessarily invoke a cause-effect relationship. However, the findings of this study can serve as the basis for a larger prospective cohort study examining a narrower profile of cytokines on a longitudinal basis.

## Conclusion

Systemic inflammation has been increasingly recognized in patients with COPD. CRP has been shown to be increased in COPD [3,4], yet many other disease states characterized by inflammation are associated with increased CRP concentrations. Our data suggests that systemic inflammation in a COPD cohort is also characterized by cytokines implicated in inflammatory cell recruitment and airway remodeling. We show associations between plasma concentrations of chemokines and IL-13 with increasing severity of disease, as measured by % FEV_1 _or % DLCO. Increasing severity of diffusion impairment is also associated with increasing G-CSF and decreasing soluble Fas concentrations. We speculate that disease characterized by disproportionate abnormalities in DLCO may be associated with peripheral markers independent of the FEV_1_. The biological plausibility of IL-13 and the discrete repertoire of inflammatory chemokines identified in our model underscore the possibility to more precisely characterize systemic inflammation of COPD.

## Abbreviations

CCL2/MCP-1, CC chemokine ligand 2/monocyte chemotattractant protein-1; CCL3/MIP-1α, CC chemokine ligand 3/macrophage inflammatory protein-1α; CCL4/MIP-1β, CC chemokine ligand 4/macrophage inflammatory protein-1β; CCL5/RANTES, CC chemokine ligand 5/regulated on activation normal T cell expressed and secreted; CCL11/eotaxin, CC chemokine ligand 11/eotaxin; CRP, C-reactive protein; CXCL8/IL-8, CXC chemokine ligand 8/interleukin-8; CXCL9/Mig, CXC chemokine ligand 9/monkine induced by interferon-γ; EGF, epidermal growth factor; EGFR, epidermal growth factor receptor; FasL, Fas ligand; FGFβ, fibroblast growth factor β; G-CSF, granulocyte-colony stimulating factor; HGF, hepatocyte growth factor; IFN-γ, interferon-γ; IL-1β, interleukin-1β; IL-2, interleukin-2; IL-2R, interleukin-2 receptor; IL-4, interleukin-4, IL-6, interleukin-6; IL-10, interleukin-10; IL-13, interleukin-13; MPO, myeloperoxidase; TNF-α, tumor necrosis factor α, TNFRI, tumor necrosis factor receptor 1, TNFRII, tumor necrosis factor receptor 2; VEGF, vascular endothelial growth factor;

## Competing interests

Frank C. Sciurba has received funding from GlaxoSmithKline and AstraZeneca in 2005 through 2006 for participation in multi-center clinical trials. He has served on advisory boards for GlaxoSmithKline and AstraZeneca. None of the other authors has any competing interests to declare.

## Authors' contributions

JSL, VK, YZ, JM, RAB, AMC and FCS participated in the design of the study. JSL contributed to the statistical analysis, interpretation of the data, and wrote the manuscript. MRR performed portions of the statistical analysis, contributed to the interpretation of the data, and revised the manuscript for important intellectual content. VK performed the statistical analysis. YZ participated in the collection of data. JM participated in the analysis of the data. RAB contributed to the analysis and interpretation of data, and revised the manuscript for important intellectual content. AMC and FCS conceived the study, contributed to the acquisition of the data, and provided important intellectual content to the manuscript. All authors read and approved the final manuscript.
